# Individual freedoms versus collective responsibility: immunization decision-making in the face of occasionally competing values

**DOI:** 10.1186/1742-7622-3-13

**Published:** 2006-09-27

**Authors:** Daniel A Salmon, Saad B Omer

**Affiliations:** 1 Department of Epidemiology and Health Policy Research, College of Medicine, University of Florida, Gainesville, Florida, USA; 2 Institute for Vaccine Safety, Johns Hopkins Bloomberg School of Public Health, Baltimore, Maryland, USA; 3 Department of International Health, Johns Hopkins Bloomberg School of Public Health, Baltimore, Maryland, USA

## Abstract

Modern public health strives for maximizing benefits for the highest number of people while protecting individual rights. Restrictions on individual rights are justified for two reasons-for the benefit of the individual or the benefit of the community.

In extreme situations there may be a need to protect the health of an individual and particularly a child; even by overriding individual/parental autonomy. However, The American Academy of Pediatrics recently concluded that "Continued (vaccine) refusal after adequate discussion should be respected unless the child is put at significant risk of serious harm (as, for example, might be the case during an epidemic). Only then should state agencies be involved to override parental discretion on the basis of medical neglect".

Many countries have compulsory immunization requirements. These laws curtail individual autonomy in order to protect the community from infectious diseases because unvaccinated individuals pose risk to the community – including vaccinated individuals (since vaccines are not 100% efficacious), children too young to be vaccinated, and persons who have medical vaccine contraindications. There are situations where there can be a real or perceived divergence between individual and community benefits of vaccination. This divergence may occasionally be based upon current scientific evidence and may exemplify the need for overriding individual autonomy. A divergence between individual and community benefits may also exist when there are ideological beliefs incongruent with vaccination or individuals are unaware of or do not accept available scientific evidence.

When the state curtails individual freedoms for the collective good, it should address several issues including the magnitude of the individual and community risk, the strength of the individual's conviction, wider and long-term consequences of restricting individual autonomy, effective risk communication, best available scientific evidence, and transparency of the decision making process.

Modern public health evolved in 19^th ^century Europe under the shadow of utilitarian ideas of Jeremy Bentham and John Stuart Mill and retains its utilitarian leanings. However, the impulse to maximize benefit for the highest number of people is counterbalanced by the Kantian threshold of a *categorical imperative*: "Act only according to that maxim by which you can at the same time will that it should become a universal law"[1] that preserves individual autonomy and emphasizes ideas such as informed consent.

Restrictions on individual rights are justified for two reasons – for the benefit of the individual or the benefit of the community. In extreme situations there may be a need to protect the health of an individual and particularly a child even by overriding individual/parental autonomy. The American Academy of Pediatrics recently concluded that "Continued (vaccine) refusal after adequate discussion should be respected unless the child is put at significant risk of serious harm (as, for example, might be the case during an epidemic). Only then should state agencies be involved to override parental discretion on the basis of medical neglect". Endemic transmission of common childhood vaccine preventable diseases, such as pertussis and varicella, may not meet this criterion of significant risk of serious harm. Due to the preventive nature of vaccines, in contrast to therapeutic treatment of existing disease, it is difficult to determine with confidence if an unvaccinated person will in fact contract disease.

Many countries have compulsory immunization requirements [2]. These laws curtail individual autonomy in order to protect the community from infectious diseases because unvaccinated individuals pose risk to the community – including vaccinated individuals (since vaccines are not 100% efficacious), children too young to be vaccinated, and persons who have medical vaccine contraindications [3,4]. In the United States (US), this reasoning is supported by several Supreme Court decisions including the landmark Jacobsen v. Massachusetts [5]. However, despite their overall societal benefit, vaccines cause severe adverse reactions in a small proportion of vaccinees. To further the societal benefits of high vaccine coverage while attempting to offset the harm for the small proportion of individuals injured by vaccines, the US established the National Vaccine Injury Compensation Program to provide no-fault compensation for vaccine injured persons [6].

There are situations where there can be a real or perceived divergence between individual and community benefits of vaccination. This divergence may occasionally be based upon current scientific evidence and may exemplify the need for overriding individual autonomy. Use of the oral polio vaccine (OPV) in the US in the early 1990s is such an example. The sustained use of OPV led to the elimination of polio in the US, with the last cases of wild polio reported in 1979. While OPV is extremely safe and effective, the vaccine very rarely caused vaccine associated paralytic polio (VAPP) resulting in 5–7 cases of VAPP annually with near universal use of OPV in the US. Once polio had been effectively controlled in the US, preventing the indigenous transmission of polio, the risks of the vaccine (VAPP) may have been greater than the risk of disease. Assuming the individual does not travel to a region where polio is still endemic, a roughly one in a million risk of VAPP is highly unlikely, but still greater than the risk of wild polio. Yet, if a substantial number of individuals were not vaccinated because of this individual risk/benefit analysis, polio would likely have been reintroduced into the US, as the disease is only a plane ride away, leading to a tragedy of the commons [7]. While this divergence in individual versus community benefits was short-lived (the US switched to the inactivated polio vaccine that can not cause VAPP), such a situation can cause a dilemma for parents, health care providers and policy makers.

A divergence between individual and community benefits may also exist when there are ideological beliefs incongruent with vaccination or individuals are unaware of or do not accept available scientific evidence. Ideological beliefs that may influence persons to forgo vaccination include religious issues (i.e. the use of cell lines from aborted fetuses to make vaccine) and a general belief that 'natural' disease is preferable to vaccines. Recent controversy surrounding association between the MMR vaccine and autism exemplify situations where some individuals *perceive *the individual risks of vaccination to outweigh the benefits. Despite carefully designed epidemiological studies [8-12] and reviews by external groups [13-17] finding no association between MMR vaccines and autism, a substantial proportion of parents maintain a belief that vaccines cause autism. From the perspective of these parents, the benefits of MMR vaccination may not outweigh the (perceived) autism risk. The community risk/benefit analysis is likewise dependent on one's knowledge base and perception of the science – undoubtedly individual vaccine refusal can lead to resurgence of disease [18,19].

Irrespective of the circumstances, when the state – acting as an agent of the society-curtails individual freedoms for the collective good, the state assumes certain responsibilities and should address the following issues. First, how great a risk does a particular health behavior entail for the individual and the community? Moreover, a distinction should be made between vaccine refusal among adults versus parental vaccine refusal, as a parent does not have an absolute right to put a child at risk even if the parent is willing to accept such risk for him or herself. Second, consideration needs to be given to the strength of the individual's conviction. The infringement upon autonomy is related to how strongly the individual opposes the intervention. Non-compliance with a public health intervention should at least be a function of conviction – not laziness. Third, policies that restrict individual rights to forgo a public health intervention must keep sight of the wider and long-term consequences of restricting individual autonomy. A draconian approach to vaccine policy risks public backlash and undermining the sustainability of vaccine programs. Fourth, public authorities should not be reflexively dismissive of concerns regarding the efficacy and safety of the intervention raised by the individuals whose rights are being restricted. Effective risk communication – including clear and coherent description of the reasons for restricting individual rights – is the responsibility of the entity imposing the restrictions. Fifth, all related decisions should be grounded in science. It is important that the decision-making process be dynamic and must be designed to be constantly informed by the emerging scientific evidence – even if the new evidence is contrary to the current scientific wisdom. Moreover, the decision making process should be transparent.

In summary, there may be situations where there is an ethically valid public health justification for restricting individual rights – both in circumstances where such actions benefit the community and in situations where the actions only benefit the individual. However, restrictions should only be placed after meeting certain conditions to ensure judicious use of this power.
